# Severity of illness and organ dysfunction scoring systems in pediatric critical care: The impacts on clinician's practices and the future

**DOI:** 10.3389/fped.2022.1054452

**Published:** 2022-11-22

**Authors:** Morgan Recher, Stéphane Leteurtre, Valentine Canon, Jean Benoit Baudelet, Marguerite Lockhart, Hervé Hubert

**Affiliations:** ^1^ Univ. Lille, CHU Lille, ULR 2694 - METRICS: Évaluation des Technologies de Santé et des Pratiques Médicales, Lille, France; ^2^ French National Out-of-Hospital Cardiac Arrest Registry, Lille, France

**Keywords:** scoring system, PICU, severity score, organ dysfunction, evaluation

## Abstract

Severity and organ dysfunction (OD) scores are increasingly used in pediatric intensive care units (PICU). Therefore, this review aims to provide 1/ an updated state-of-the-art of severity scoring systems and OD scores in pediatric critical care, which explains 2/ the performance measurement tools and the significance of each tool in clinical practice and provides 3/ the usefulness, limits, and impact on future scores in PICU. The following two pediatric systems have been proposed: the PRISMIV, is used to collect data between 2 h before PICU admission and the first 4 h after PICU admission; the PIM3, is used to collect data during the first hour after PICU admission. The PELOD-2 and SOFApediatric scores were the most common OD scores available. Scores used in the PICU should help clinicians answer the following three questions: 1/ Are the most severely ill patients dying in my service: a good discrimination allow us to interpret that there are the most severe patients who died in my service. 2/ Does the overall number of deaths observed in my department consistent with the severity of patients? The standard mortality ratio allow us to determine whether the total number of deaths observed in our service over a given period is in adequacy with the number of deaths predicted, by considering the severity of patients on admission? 3/ Does the number of deaths observed by severity level in my department consistent with the severity of patients? The calibration enabled us to determine whether the number of deaths observed according to the severity of patients at PICU admission in a department over a given period is in adequacy with the number of deaths predicted, according to the severity of the patients at PICU admission. These scoring systems are not interpretable at the patient level. Scoring systems are used to describe patients with PICU in research and evaluate the service's case mix and performance. Therefore, the prospect of automated data collection, which permits their calculation, facilitated by the computerization of services, is a necessity that manufacturers should consider.

## Introduction

Mortality in pediatric intensive care units (PICU) is approximately 2.4% in the United States (2014–2019) (1) and 3.5% in UK (2017–2019) (2), representing a “gold standard” judgment criterion. This gold standard criterion is established either at PICU discharge (3, 4) or at hospital discharge (5). Therefore, admission severity scores were developed and validated, considering the physiological parameters collected during the first hours of hospitalization in the ICU to quantify the patients' health status on admission to the ICU. In pediatric intensive care, these prognostic or predictive scores are established independently of the diagnosis, considering the heterogeneity of the populations regarding age, particularly to make outcome assessment between PICUs more objective (6, 7).

**Figure 1:**
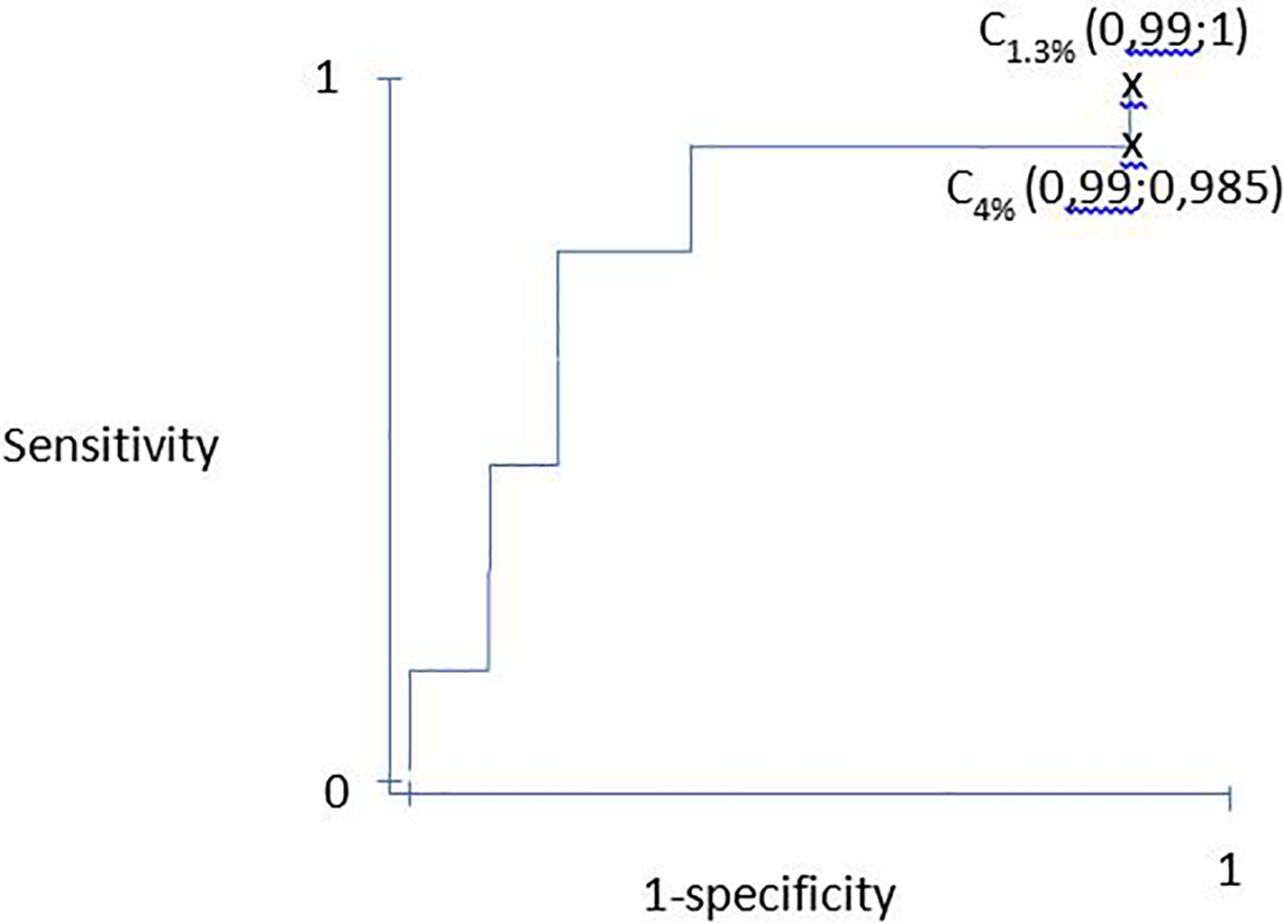
ROC curve example based on tableau 1. ROC, Receiver operating curve.

Simultaneously, during the PICU stay, the description and quantification of organ dysfunction (OD) have been important since the 1990s. Indeed, the frequency of these ODs is related to mortality (8, 9). These ODs may exist at admission or during their stay in the ICU. First, formal criteria for OD were initially proposed by Wilkinson in 1986 (9), Proulx in 1996 (8), and Goldstein in 2005 (10) to maximize multiple organ dysfunction syndrome (MODS) detection (6). In 2022, the Pediatric Organ Dysfunction Information Update Mandate (PODIUM) expert panel summarized data characterizing single and multiple OD and derived contemporary criteria for OD (11–13). A consensus was reached for a final set of 43 criteria for MODS. The PODIUM criteria for MODS are limited by available evidence and will require validation; however, they provide a contemporary foundation for researchers to identify and study single and multiple OD in critically ill children (13). Second, OD scores, considering physiological parameters reflecting the main ODs, have been developed and validated initially to maximize the description of the clinical course and severity of illness in ODs during the ICU stay and not as predictive tools of mortality (6). Therefore, in children, the daily collection of Pediatric Logistic Organ Dysfunction (PELOD) data showed that the mortality of patients was greater than 50% if there was a worsening score between day 1 (D1) and D2 and between D2 and D5 (3). The following “target days” corresponding to the days of PELOD score collection for which the score is most related to mortality during the stay in the ICU (significant mortality hazard ratio for each of these target days) were determined: Day (D)1, D2, D5, D8, D12, D16, and D18 (3). Therefore, mortality is the gold standard for developing and validating OD scores. However, it has been established that once constructed (vs. mortality), these OD scores become a primary or secondary endpoint, independently of mortality (14).

Therefore, this review aims 1/ to provide updated state of the art of severity scoring systems and OD scores in pediatric critical care, 2/ to describe the impacts of scoring systems on clinicians' understanding of practices, and 3/ to provide the usefulness, limits, and implications for the future of the scores in PICU.

## An updated state-of-the-art of severity scoring systems and OD scores

### What severity scores are available in pediatric intensive care?

In pediatric intensive care, the interest in assessing severity is reinforced by the heterogeneity of the population (from newborns to adolescents) and the diagnoses encountered. However, the following two “systems” have been proposed for the population, from newborns (excluding premature babies) to adolescents:

1. The Pediatric Risk of Mortality (PRISM) score system can be used for term newborns to adolescents. The first version in 1984, named “Physiologic Stability Index,” included 24 variables (15). In 1988, Pollack et al. published a new version of the PRISM score (known as PRISM II), which included 14 variables in the first 24 h in the PICU (16). In 1996, a new adaptation, the PRISM III score, which included 17 variables, was published (17). PRISM III score data were collected in the first 12 or 24 h after admission to the ICU. The most pathological value for each variable was considered during this study. The PRISM III score's strengths are that it has been validated on a sample of 11,165 patients from 32 PICUs in the United States and that it is adapted periodically from an American PICU data collection site (17). Additionally, it is possible to calculate the PRISM III score independently of the probability of death. The following are the two main limitations to the PRISM III score: (1) the relatively long period of data collection of the PRISM III score (12–24 h) reflects not only the initial severity but also the management during this period; (2) the coefficients of each variable necessary to calculate the probability of death are not in the public domain. Furthermore, the PRISM IV version was published in early 2016 (5). Data were collected between 2011 and 2013, including a prospective cohort of 10,078 ICU admissions (newborn to 18 years) from seven North American services. The outcome was live/dead at discharge after the first pediatric intensive care admission. The collection period was 2 h before PICU admission (by emergency mobile service) and the first 4 h after PICU admission. The variables collected and categorized were identical to the PRISM III scores (17). The equation for calculating the probability of death is free (5). It considers age, origin, cardiac arrest in the previous 24 h, cancer, low-risk main dysfunction on admission, and scores according to the categories of neurological and non-neurological variables (5).
2. The Pediatric Index of Mortality (PIM) score system can be used for a term neonate until 16 years. The first version of the PIM score in 1997 included eight variables collected during the first hours after admission to a PICU (18). In 2003, PIM2 was developed and validated in 20,787 patients in Australia and the United Kingdom and included 10 variables (19). PIM3, which was developed in 2013 from a sample of 53,112 patients, consists of 10 variables (4). The variables collected were identical between the PIM2 and PIM3 score versions. However, the variable items were reorganized (low-risk and high-risk diagnoses in PIM2 were redistributed into low-risk, high-risk, and very high-risk diagnoses in PIM3). The following are the strengths of the PIM3 score: the large size of the validation population, the quantitative assessment as early as 1 h after admission to the ICU, the publication of the coefficients for each variable, and the probability of the death equation. However, the main limitation of the PIM3 score is that it was constructed only from the variables of the PIM2 score (without testing new potential variables). A study in 17 Italian PICUs, including 11,109 patients, showed good performance of the PIM3 (20).

These two “systems” have different characteristics that can guide the choice of one or the other, depending on the priorities chosen. However, some authors have mentioned that the available scores are inappropriate for developing countries (21).

### What are OD scores currently available?

The PELOD scores (1999 and 2003) contained 6 ODs and 12 variables. The main limitation of the PELOD score is that it presents unobservable values on a discrete scale from 0 to 71. Therefore, there are difficulties in interpretation when calculating the means or medians of the PELOD scores (22, 23). The PELOD-2 score, which was developed and validated in 2013 from a sample of 3,761 patients from 15 European services, has five ODs and 10 variables (24). The main difference between the two versions is the deletion of the hepatic OD in the PELOD-2 score and the replacement of systolic blood pressure and heart rate from the PELOD score by mean arterial pressure and lactatemia, respectively, in the PELOD-2 score. For the PELOD-2 score, discrete values between 0 and 33 points were possible. Therefore, the collection of the PELOD system is based on a daily collection over a 24-h period, starting from the admission schedule. The most relevant collection days (so-called “target days”) for predicting mortality can be determined for both PELOD and PELOD-2 scores (3, 25). Equations for calculating the probabilities of death for the PELOD system have been published (3, 25).

The Pediatric Multiple Organ Dysfunction Score (P-MODS) was developed and validated in a single United States service, including 6,456 patients in 2005 (26). The P-MODS score contains five ODs (cardiovascular, respiratory, renal, hematologic, and hepatic); however, it excludes neurological dysfunction. Each of these five ODs is characterized by biological variables. An equation for calculating the probability of death has not been previously published (26). The P-MODS score has never been the subject of published external validation.

In 2017, the pediatric sequential organ failure assessment (pSOFA) was published to perform the first assessment of Sepsis-3 in critically ill children. The pSOFA score was developed by adapting the original SOFA score using two approaches. First, the original SOFA score's age-dependent cardiovascular and renal variables were modified using validated cutoffs from the PELOD-2-scoring system. Second, the respiratory sub-score was expanded to include the SpO2:FiO2 ratio as an alternative surrogate for lung injury. Sepsis-3 definitions were assessed in children with confirmed or suspected infection using the pSOFA score (27). However, the pSOFA score does not allow the calculation of the probability of death.

Recently, pediatric “quick” scores with three variables (ranging from 0 to 3) have been proposed. The pediatric-age-adapted-quick-SOFA (qSOFA) (28) and quick-PELOD-2 (qPELOD-2) (29) have been developed in different settings. The performances of these two scores varied according to the case mix of the population (30–32).

### Calculation of the probability of death

The probability of death can be calculated in the following two different ways depending on the scores:

1. The PRISM and PELOD scoring systems calculated the score value for each patient. This score was transformed into the probability of death using an equation (5, 23, 24). This equation is freely available for the PELOD system (23, 24), PRISM (16), and PRISM IV (5) scores, but requires a license for the PRISM III score.
2. The PIM system (PIM, PIM2, and PIM3 scores) does not allow the calculation of the value of the score but allows direct calculation of the probability of death from the variables (4, 18, 19).

### Quality of scoring systems in intensive care

Severity scoring systems have several strengths before they can be used routinely. The included variables should be relevant to medical recommendations, usual, objective, easy to collect, rapid, and early after admission. Therefore, the prognostic score should have good intra- and inter-observer reproducibility and the ability to detect fine variations in severity between patients (sensitivity to change), which should be validated after comparison with other traditionally recognized prognostic scores or indices, “acceptable” to the patient, simple to use for the physician, of low cost, and “feasible” in any department likely to apply it (6). These quality criteria justify regularly updating the severity and OD scores (33).

## Pediatric scoring systems: impacts on understanding for the clinicians in 2022

### Scores used in PICU should help clinicians answer the following questions

A. “Are the most severe patients dying in my department?”
B. “Does the overall number of deaths observed in my department consistent with the severity of illness of patients?”
C. “Does the number of deaths observed by severity level in my department consistent with the severity of illness of patients?”

The statistical tools used to evaluate the scores' performance and answer the three questions are described below.

A. “Are the most severely ill patients dying in my department?” Discrimination in the scores allowed us to answer the first question.

Admission scores for patients who survive should be lower than those observed for patients who die. Discrimination can be assessed either from the score value or the probability of death calculated from the score. Indeed, the transformation from the score to the probability of death is a monotonic (logarithmic) function, which does not change the ranking order between the score value and the likelihood of death. Therefore, discrimination is a measure of the ability of a score to “assign” lower score values or probabilities of death to patients who will live and to “assign” higher score values or probabilities of death to patients who will die. Moreover, discrimination only considers the ranking of the score or the probability of death, independent of the values of the scores or probabilities of death obtained. Therefore, it is theoretically possible that all patients in a department are ideally classified between living and dead based on a range of probability of death between 1% and 13%. In this example, the score would be perfectly discriminating if all the surviving patients were classified between 1% and 4% and the deceased patients were between 5% and 13%. In contrast, no patient would have a probability of death higher than 13%. Therefore, we perceive a limit to this discrimination criterion because the value of the score (or probability) obtained is not considered (but only the classification of the values).

Discrimination was evaluated by calculating the area under the receiver operating characteristic (ROC) curve. Therefore, the ROC curve was obtained by successively varying the thresholds of the score and calculating the sensibilities and specificity for each threshold. The ROC curve represents the variation in (1-specificity) as a function of the score's sensitivity (Table 1). The area under under the curve (AUC) is interpreted as follows: an area under the ROC curve equal to 0.50 means that the score is not more discriminating than chance, an area between 0.70 and 0.79 is considered correct, an area between 0.80 and 0.89 is considered good, and an area >0.90 excellent (34, 35). A confidence interval is calculated, the upper limit of which cannot be greater than 1 (36). Good discrimination allowed us to interpret that patients with the highest probability of death died more frequently than patients with the lowest probability of death. Hence, the most severe patients died in my department. Furthermore, the Youden index can be combined with discrimination to determine the best cutoff to discriminate survivors from non-survivors (37).

**Table 1:** Example of discrimination for a score on a population (500 patients).

Patients	Value score (increasing order)	Probability of death, value; (%)		At PICU discharge (alive = 0/died = 1)	
1	5	0.010;	(1%)		0
2	6	0.012;	(1.2%)	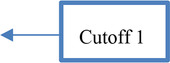	0
3	7	0.014;	(1.4%)		1
4	7	0.014;	(1.4%)		0
5	10	0.035;	(3.5%)	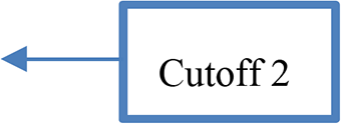	0
6	12	0.043;	(4.3%)		1
…..	…..	…..	…..		…..
499	30	0.957;	(95.7%)		1
500	31	0.968;	(96.8%)		1
					Sum = 69
**Example 1: cutoff 1: 1.3%: calculation for sensitivity and specificity:**					
	**Death**	**Alive**			
Probability of death > 1.3%	69	429	498		
Probability of death < 1.3%	0	2	2		
	69	431	500		
Sensitivity = 69/69 = 1; specificity = 2/431 = 0.0046					
Couple of point (1- specificity; sensitivity) for cutoff 1.3%: (0.99; 1)					
**Example 2: cutoff 2: 4.0%: calculation for sensitivity and specificity:**					
	**Death**	**Alive**			
Probability of death > 4.0%	68	427	495		
Probability of death < 4.0%	1	4	5		
	69	431	500		
Sensitivity = 68/69 = 0.985: specificity = 4/431 = 0.0092					
Couple of point (1- specificity; sensitivity) for cutoff 4.0%: (0.99; 0.985)					
PICU: pediatric intensive care unit

B. “Does the overall number of deaths observed in my department consistent with the severity of patients?” The standardized mortality ratio answered the second question:

The Standardized Mortality Ratio (SMR) is defined as the ratio of the number of observed deaths divided by the number of predicted deaths during a period (38). The number of observed deaths was the number of deaths in the population under study (69 in the example in Table 2). The number of predicted deaths was obtained by summing all probabilities of death for the patients in the population (74.2 in the example in Table 2). Notably, when the SMR was less than one, the number of observed deaths was less than the number of predicted deaths (Table 2). When SMR was greater than 1, the number of observed deaths was greater than the number of predicted deaths. A formula for calculating the confidence interval for the SMR exists. If the SMR confidence interval includes 1, the difference between the number of observed deaths and the number of predicted deaths is insignificant (Table 2). If the confidence interval excludes 1, the difference between the observed and predicted numbers of deaths is significant.

**Table 2:** Example of standardized mortality ratio (SMR) in a population with 500 patients for any score.

Patients	Value score (increasing order)	Probability of death, value; (%)		At PICU discharge (alive = 0/died = 1)
1	5	0.010;	(1%)	0
2	6	0.012;	(1.2%)	0
3	7	0.014;	(1.4%)	1
4	7	0.014;	(1.4%)	0
5	10	0.035;	(3.5%)	0
6	12	0.043;	(4.3%)	1
…..	…..	…..	…..	…..
499	30	0.957;	(95.7%)	1
500	31	0.968;	(96.8%)	1
Sum		74.2		69
		Predicted deaths		Observed deaths

SMR = Observed deaths/Predicted deaths = 69/74.2 = 0.93 (95% confidence interval: 0.87–1.02).

PICU, pediatric intensive care unit.

Therefore, it is possible to determine whether the total number of deaths observed in my service over a given period is in adequacy with the number of deaths predicted, by considering the severity of patients on admission.

C. “Does the number of deaths observed by severity level in my department correspond to the severity of the patients in my department?” Calibration of the scores allowed us to answer this question.

Calibration measures how well the predicted mortality matches the observed mortality by severity level at PICU admission. The severity levels can be defined in several ways. Generally, 10 groups (or classes) of severity levels are considered: 0%–10%, 10%–20%, etc., and patients are classified according to their probability of death (39). This classification can lead to an imbalance in the number of patients per subgroup (more patients in some subgroups and very few in others). Therefore, sorting the patients in the ascending order of their probability of death is also possible, and considering between 5 and 10 groups with the same number of patients per group:10 groups correspond to the deciles of predicted probabilities (Table 3) (40). In each group, two predicted numbers were calculated: the number of predicted deaths (which corresponds to the sum of the predicted probabilities of death for all individuals in the group) and the number of predicted alive patients (=1-sum OF “predicted probabilities of deaths”). When considering deaths, two factors are generally expected: (1) The number of observed deaths and predicted deaths were lower in subgroups with a low probability of death than in those with a high probability of death. (2) In each group, the number of observed deaths was close to the number of predicted deaths. Hosmer-Lemeshow's goodness-of-fit statistical test was used to perform an overall comparison of observed (deaths and alive) vs. predicted using the chi-square test (40). The *P*-value was deduced after defining the number of degrees of freedom (ddl). The number of ddls was equal to the number of subgroups −2 (8 in our example) for score development. The number of ddls was equal to the number of groups used for score validation (35). Because it is expected that there will be no difference between the number of observed deaths and the number of predicted deaths, the calibration of the score is good (or adequate) when the test is insignificant at the 5% level: a *P*-value greater than 0.05 (34). Calibration is a demanding test; if the number of observed deaths is very different from the number of predicted deaths in a single group, the score calibration is probably poor (*P* < 0.05) (Table 3) (39). Furthermore, when a score's calibration in a population is good, it can be concluded that the number of deaths observed is close to the number of deaths predicted. This adequacy is a function of the patients' severity level. Therefore, it is possible to determine whether the number of deaths observed according to the severity of patients at PICU admission in a department over a given period is in adequacy with the number of deaths predicted, according to the severity of the patients at PICU admission.

**Table 3:** Example of Hosmer–Lemeshow goodness of fit test.

Decile of risk	Survival	Survival	Non-survival	Non-survival	Total
Hospital mortality	Observed	Expected	Observed	Expected	
**APACHE II score on day 3**					
1	341	341,392	14	13,608	355
2	327	330,766	28	24,234	355
3	329	321,526	26	33,474	355
4	318	311,866	37	43,134	355
5	302	299,902	53	55,098	355
6	275	284,631	80	70,369	355
7	269	266,116	86	88,884	355
8	239	244,417	116	110,583	355
9	207	211,18	148	143,82	355
10	148	143,203	206	210,797	354
**APACHE II score on day 1**					
1	565	581,87	65	48,13	630
2	562	561,981	68	68,019	630
3	563	548,176	65	81,824	630
4	534	535,885	96	94,115	630
5	542	524,26	88	105,74	630
6	519	512,451	111	117,549	630
7	517	499,186	113	130,814	630
8	488	483,031	142	146,969	630
9	426	458,489	204	171,511	630
10	382	394,67	251	238,33	633

The results indicated that there was no significant difference between the predicted mortality and the actual mortality (*X*^2^ = 6.198, *P* = 0.625), and the consistency of the predicted mortality rate and the actual rate was 79.4%, suggesting that APACHE II score-based predictive model on day 3 has a good calibration ability to predict hospital mortality. However, the APACHE II score on day 1 had poor calibration in predicting the hospital mortality rate of the patients (*X*^2^ = 294.898, *P* < 0.001) (69).

### Adaptation of the scores

Severity scores establish the probability of death at PICU admission (within the first 24 h after admission). Therefore, discrimination and calibration tests are usually used to validate these scores. In contrast, OD scores are intended to assess OD during ICU stay and are not predictive of mortality (6). Thus, only the discrimination criterion is often necessary to evaluate the performance of OD scores. Some authors have tested or compared severity and OD scores as prognostic tools and performed calibration calculations for both types of scores. OD scores are frequently relevant for this purpose (41).

Severity or OD scores were developed and validated in the general PICU population. It is expected that the application of this score to a new population in a different location (external geographical validation) will allow the confirmation of this score in this new population. However, the external validation of scores tested on a new population generally has mostly poor calibration (42). The explanation is not a change in the performance of one team compared with another; however, it is essentially different recruitment of services due to regional or national variations in the organization of care (43, 44). Therefore, the initial equation does not allow the reliable calibration of an external population. It is necessary to evaluate the calibration of scores by adapting (or customizing) the score to the new population tested (45), even if this adaptation compromises comparability with the original population (20). There are three levels of customization as follows. (1) First-level customization, which involves assigning a global correction coefficient to the calculated score to adapt it to the new population (but without modifying the variables or the coefficients assigned to each variable) (45). Unfortunately, many authors ignore this step, use the severity and OD scores as predictive tools, and hastily conclude that a score is poorly calibrated without performing this first level of adaptation. However, this first-level customization does not address all the problems of updating. Notably, care improvement has decreased ICU mortality over the years. Therefore, the coefficients assigned to each variable in the equations to calculate the probability of mortality lose accuracy. (2) The second-degree customization comprises each variable and recalculates the coefficient assigned to each variable by considering the mortality of the new population tested. (3) Finally, the scores were established at a specific time, considering the available clinical and biological assessment variables. Over the years, few assessment tools have been used (e.g., blood-drawn PaO2), although other more relevant ones have appeared (lactatemia, among others). The third-degree customization is a complete update of the score, comprising updated variables included in the scores and calculating the coefficient of each variable of the new score. The score versions (PIM2, PIM3, PRISM III, PRISM IV, PELOD, and PELOD-2) were also modified (38).

## Pediatric-scoring systems: use and impacts on the future

### Objective assessment of patient severity and OD

Assessing patient severity and OD is the primary goal in the ICU. Therefore, the physician in charge of the patient considers the clinical and paraclinical factors to achieve this aim. Notably, the probability of death cannot be used for an individual diagnostic or therapeutic decision in managing the patient. Specifically, when a decision to limit therapy was taken for each patient in a group of 10 patients, each with an 80% probability of death, all 10 patients would die. However, it is “predictable” that among these 10 patients, each with an 80% probability of death, only two patients (unidentifiable by the calculation) would survive. Therefore, the likelihood of death is not interpretable at the patient level (33).

### Description of recruitment and criteria for inclusion in the studies

The severity and OD scores facilitate patients' description, which is included in the studies for characterizing the study population (6). The use of severity score as an inclusion criterion in trials is highly controversial. Additionally, the severity and OD scores should not be used for this purpose (33). However, stratification based on severity, which is assessed by scores, should be preferred in designing outcome analysis.

### Tools for randomized trials

PRISM, PIM, PELOD, and pSOFA have been used to study the comparability of groups in randomized trials. In the pediatric transfusion requirements in a PICU (TRIPICU) study to determine the best transfusion threshold of packed red blood cells, the PRISM score was comparable after randomization between the “liberal strategy: transfusion at a threshold of 9.5 g/dl” group and the “restrictive strategy: transfusion at a threshold of 7g/dl” group (46). Additionally, in the same pediatric study, the primary endpoint was new or progressive organ failure (MODS). Conversely, the secondary endpoint was the PELOD score. Furthermore, severity and OD scores can also be used as adjustment criteria in clinical trials.

### Evaluation of recruitment and performance of services

Severity and OD scores can assess the evolution of recruitment and determine the SMR in a service. Similarly, it is possible to perform and compare this approach in several services. However, the previous application has some limitations. Therefore, when the general severity scores are ideally constructed independently of patient diagnoses and applied to all intensive care populations, it appears that the recruitment or organization of the services (cardiac surgery in one center, neonatal orientation in another center, the policy of eligibility or discharge, and the existence of a downstream continuous monitoring unit, among others) modifies the value of the SMR and that an adaptation of the scores could be necessary to facilitate comparability (47, 48).

### Impacts on the future

#### Quality of life scores in the ICU

Reducing mortality is the primary objective of PICU development. In Australia, the observed mortality rate in PICUs was 4.7% in 1996 (*n* = 1161) (49). The American Registry of PICU reported a mortality rate of 3% between 2005 and 2008 (*n* = 80,739 patients) (50). Additionally, a study comparing French and English populations over the period 2006–2007 showed mortality rates of 7.4% (*n* = 5602, French patients) and 4.9% (*n* = 20,693, English patients), respectively (47). These international variations in mortality rates, which were established in countries with similar levels of development, can probably be explained by different cases mixed and including or excluding intermediate care units. However, there has been a progressive reduction in mortality in all countries (49). Therefore, Pollack et al. developed and validated a predictive tool established at admission, considering a ternary judgment as a criterion: alive without new morbidity, alive with morbidity, or death (50). Additionally, morbidity status was quantified using the scale developed by the same team in 2009 (functional performance scale), which considers six domains (consciousness, sensory, communication, motor, feeding, and breathing) with a quantification between 1 (normal) and 5 (very severe dysfunction) for each domain (51).

The next step is quantifying the medium-term morbidity after discharge from the ICU (52). A review by Aspesberro et al. identified four quality of life assessment scales that can be used in pediatric resuscitation trials: the Pediatric Quality of Life Inventory version 4.0 (Peds QL 4. Zero Generix core scale) (53), KIDSCREEN-27 (54), KINDL, and Child Health Questionnaire-Parent Form (CHQ-PF28) (52), for children aged 2–18, 8–18, 6–18, and 5–18 years, respectively. In 2019, Matics et al. proved that the maximum pediatric SOFA and PELOD-2 scores during critical illness had a good to excellent performance in predicting new morbidity or mortality for approximately 3 years after critical illness. Therefore, using these MODS scores may be helpful in the prognosis of longitudinal functional outcomes in critically ill children (55).

#### Future impacts on trajectories of PICU patients and organization

Recently, novel indicators have been proposed to assess the severity of disease trajectories. Interestingly, the criticality index model estimates the probability of ICU care for a 6-h duration using a calibrated, deep neural network. The criticality index exhibited strong validity, which reflects the expected clinical course for five different patient groups (56). Additionally, a recurrent neural network was trained to continuously generate individual severity-of-illness scores from electronic medical record data by predicting the risk of ICU mortality. Interestingly, it could process hundreds of variables from the electronic medical record (EMR) and integrate them dynamically as the measurements become available. The results provided an accurate, continuous, and real-time assessment of a child's condition in the ICU (57). However, for clinical decision-support tools to change outcomes, clinicians should be willing to trust them. The “Black box” models are less likely to be trusted. Additionally, approaches to improve interpretability exist in the machine learning literature, although they are rarely used in biomedicine (58, 59).

Thus, EMR represents an extremely important element of discussion for the future. The challenges of the EMR are very well described (60, 61). The development of the EMR must be done through a collaboration between engineers and pediatric intensive care physicians. The issues should not be restricted to the computerization of the scoring system, but should aim at the development of tools for personalized medicine, by integrating the collective learnt experience. The deployment of such a tool has already been proposed in a singular but adapted way in pediatric intensive care (62–65). The computer tool development era has been around for 30–40 years. The era of daily benefits for patients through practical and personalized applications to optimize medical care must be accelerated.

Moreover, these results should be analyzed collectively, understanding the local characteristics, to prevent erroneous interpretations. Therefore, the need for annual national monitoring of medical and medico-economic activities has led to the development of national networks of PICUs in many industrialized countries: North America (Virtual PICU Performance System, “VPS” https://portal.myvps.org/) (66), Great Britain (Paediatric Intensive Care Audit Network, “PICAnet” http://www.picanet.org.uk/) (2), and Australia—New Zealand (Australian and New Zealand pediatric Intensive Care Society, “ANZPICS” http://www.anzics.com.au/pages/CORE/ANZPICR-registry.aspx) (67), and the PICU Registry in France (PICURe) (68). These pediatric intensive care collective networks aim to build a database. The first objectives of these databases are medico-economic by assessing supply and demand at local, regional, and national levels to improve planning of health care strategies, and by monitoring the disease epidemiology of services. The second objective concerns clinical aspects by quantifying outcome indicators such as mortality, morbidity, and adverse events, and by promoting multicenter clinical studies.

## Conclusion

Since scores in pediatric intensive care are constantly evolving, understanding their updating is necessary, and the interpretation limits of their results should be sufficiently known, both for the clinician in his management (individual prognosis and inclusion in protocols, among others) and concerning performance analysis (need for regular adaptations before any conclusions). Therefore, the prospect of automated data collection that enables their calculation, facilitated by the computerization of services, is a necessity that manufacturers should consider (60, 62). There is still a long way to go and we must not lose sight of the fact that informatics must be at the service of medicine and not the other way around.
